# Drug use disorder and risk of incident and fatal prostate cancer among Swedish men: a nationwide epidemiological study

**DOI:** 10.1007/s10552-021-01513-2

**Published:** 2021-11-07

**Authors:** Disa Dahlman, Xinjun Li, Casey Crump, Jan Sundquist, Kristina Sundquist

**Affiliations:** 1 Center for Primary Health Care Research, Department of Clinical Sciences, Lund University/Region Skåne, Malmö, Sweden; 2 Departments of Family Medicine and Community Health and of Population Health Science and Policy, Icahn School of Medicine at Mount Sinai, New York City, NY USA; 3 Center for Community-Based Healthcare Research and Education (CoHRE), School of Medicine, Shimane University, Matsue, Japan

**Keywords:** Prostate cancer, Drug abuse, Mortality, Sweden, Epidemiology

## Abstract

**Purpose:**

Prostate cancer is the second most common cancer in men and a leading cause of cancer mortality worldwide. Men with drug use disorders (DUD) may potentially be at high risk for prostate cancer mortality because of delayed diagnosis and/or undertreatment. In this study, we aimed to investigate prostate cancer incidence, mortality, and stage at time of diagnosis among men with DUD compared to the general male population in Sweden.

**Methods:**

We performed a follow-up study based on Swedish national register data for the period January 1997–December 2016. The study was based on 1,361,532 men aged 50–75 years at inclusion, of whom 9,259 were registered with DUD. Cox regression analysis was used to compute adjusted hazard ratios (HRs) for incident and fatal prostate cancer, and cancer stage at time of diagnosis, associated with DUD.

**Results:**

DUD was associated with a slightly increased risk of incident prostate cancer (HR: 1.07, 95% confidence interval [CI] 1.00–1.14, *p* = 0.048) and substantially higher risk of fatal prostate cancer (HR: 1.59, 95% CI 1.40–1.82, *p* < 0.001), adjusted for age, socioeconomic factors, and comorbidities related to tobacco smoking and alcohol use disorder. No association was found between DUD and prostate cancer stage at diagnosis.

**Conclusions:**

Men with DUD have an increased risk of fatal prostate cancer, possibly related to undertreatment in this patient population. Our findings should raise attention among medical staff and decision-makers towards a disadvantaged group of men in need of easily accessible prostate cancer evaluation and treatment.

**Supplementary Information:**

The online version contains supplementary material available at 10.1007/s10552-021-01513-2.

## Introduction

Prostate cancer is the second most common cancer in men and a leading cause of cancer mortality worldwide [1, 2]. Men with drug use disorder (DUD) may have a higher risk of prostate cancer incidence and mortality because of delayed diagnosis and/or undertreatment. This issue has rarely been examined and is important because DUD has been associated with a range of adverse health outcomes including drug-related [3, 4] and non-drug-related mortality [4–7], breast cancer incidence and mortality [7], blood-borne infections [8, 9], and liver disease [10]. The substantial physical morbidity associated with DUD may be partially related to healthcare seeking barriers [11–13], poor health literacy [14], psychiatric comorbidity [15–17], and under-utilization of cancer screening [18–21].

The health risks among people with DUD may further be affected by low socioeconomic status (SES). Several SES factors, for example neighborhood deprivation, being unmarried, or having low income or educational attainment, have been associated with increased prostate cancer risk and worse prostate cancer prognosis [22]. Lifestyle factors such as tobacco smoking and negative alcohol-related consequences are overrepresented among people with low SES [23, 24] or DUD [25–29], and are also identified as risk factors for prostate cancer and poor prognosis in some studies although the associations are heterogeneous [30–32].

It is thus possible that men with DUD have a delayed diagnosis and higher mortality in common cancers, including prostate cancer, which is the second most commonly diagnosed cancer among men globally [1]. In Sweden, a country with tax-financed healthcare that is strongly subsidized for the individual [33], prostate cancer is the most common cancer among men with approximately 10,000 men being diagnosed annually [34]. There is no national screening program for prostate cancer in Sweden. Nevertheless, approximately half of the Swedish male population age 50–70 have had a prostate specific antigen (PSA) test, which is offered free of charge to men over 50 years on their own initiative [34].

Despite the importance of understanding whether men with DUD have a higher prostate cancer incidence or mortality, research on this subject is very sparse. It has been shown that cannabis use is associated with higher incidence of prostate cancer [35], and that substance use disorder (SUD; alcohol included) is associated with adverse health outcomes in men with advanced stage prostate cancer [36, 37]. There is limited evidence regarding potential mechanisms linking substance use and prostate cancer, and substance-specific, biological mechanisms [35] as well as behavioral mechanisms such as healthcare seeking and treatment compliance [36] have been suggested in the scientific literature. Considering the high prevalence of unmet healthcare needs and delayed healthcare seeking among people with DUD, we hypothesized that DUD would be associated with an increased prostate cancer mortality, and more advanced cancer stage at the time of prostate cancer diagnosis. In the present study, we utilize nationwide population and healthcare registries with highly comprehensive individually linked data to examine prostate cancer incidence, mortality, and stage at the time of diagnosis among men with DUD compared to the general male population in Sweden, after adjusting for potential confounders, including SES.

## Methods

### Data sources

This retrospective cohort study was based on Swedish national register data. We constructed a dataset by linking data from the Swedish National Patient Register (NPR) for inpatient care (1964–2016) and outpatient care (2001–2016), the Swedish Cancer Register (1958–2016), the Total Population Register, the Swedish Cause of Death Register (1961–2016), the Swedish Prescribed Drug Register (2005–2016), the Crime Register (1973–2016), and the Suspicion Register (1995–2016). The national 10-digit civic registration number, which is assigned to each person in Sweden upon birth or immigration to the country, was used for register linkage. The national 10-digit civic registration number was replaced by a serial number to ensure the confidentiality of all individuals. The registers contain, e.g., individual-level data on age, sex, education, hospital admissions, dispensed drugs, and prostate cancer diagnoses on a nationwide basis, including the study population of men aged 50–75 years in Sweden.

Ethical approval for the study was obtained from the Regional Ethical Review Board in Lund (File Number 2012/795).

### Participants

A total of 1,371,268 men in Sweden were 50–75 years of age on 1 January 1997. The age range was motivated by the very low incidence of prostate cancer in men < 50 years, and the estimated survival time in the study sample (the life expectancy among Swedish men is approximately 80 years). Those with a diagnosis of prostate cancer between 1 January 1991, and 31 December 1996 (i.e., with an earlier diagnosed cancer) were excluded (*n* = 9736), leaving 1,361,532 men for inclusion in the study.

### Exposure

*Drug use disorders (DUD)* were identified any time during the study period, and defined as a registration in the following registries: the NPR by relevant ICD-10 codes (F10–F19: mental and behavioral disorders due to psychoactive substance use, except those due to alcohol or tobacco); the Suspicion Register, which records suspected crimes related to drug use by codes 3070 (driving under the influence of drugs), 5010 (drug possession), 5011 (drug use), and 5012 (drug possession and use); and the Crime Register, which records convictions by references to laws covering narcotics (law 1968:64, paragraph 1, point 6) and drug-related driving offenses (law 1951:649, paragraph 4, subsection 2 and paragraph 4A, subsection 2). DUD was also identified in individuals (excluding any other cancer patients) in the Prescribed Drug Register who had filled prescriptions for hypnotics and sedatives (Anatomical Therapeutic Chemical [ATC] Classification System N05C and N05BA) or opioids (ATC: N02A) in average dosages of more than four defined daily doses a day for 12 months.

### Outcome variables

*Incident prostate cancer* was defined as a diagnosis of prostate cancer according to the 7th revision of the International Classification of Diseases (ICD-7 177) in the Swedish Cancer Register during the study period (1997–2016). The Swedish Cancer Register has transferred all cancer ICD codes into ICD-7.

*Fatal prostate cancer* was defined as a death attributed to prostate cancer as the primary cause according to the 10th revision of the ICD (ICD-10 C61) in the Swedish Cause of Death Register during the study period (1997–2016).

*Prostate cancer stage* 0–IV was obtained from the Swedish Cancer Register based on the TNM classification. TNM refers to Tumor (tumor characteristics, including size), Nodes (spread to nearby lymph nodes), and Metastases (spread to other parts of the body). Prior to statistical analysis, cancer stage was dichotomized into stage I-III and stage IV (i.e., metastasized prostate cancer). In the additional analysis, each cancer was only included once. Prostate cancer cases with tumor stage were included as outcomes.

### Covariates

*Age* at entering the cohort was recorded as a continuous variable at 1 January 1997.

*Educational attainment* as of 1 January 1997 was categorized as ≤ 9 years (partial or complete compulsory schooling), 10–11 years (partial or complete secondary schooling), and 12 ≤ years (some or completed college and/or university studies). Prior to the statistical analysis, educational attainment was dichotomized into “ < 12 years” and “12 ≤ years.”

*Social welfare*, received from social services, was defined as Yes (received) and No (not received).

*Marital status* as of 1 January 1997 was classified as married/cohabiting, unmarried, divorced, or widowed. Prior to the statistical analysis, marital status was dichotomized into “married/cohabiting” vs. “not married/cohabiting” (including all unmarried, divorced, and widowed men).

*Region of residence* was categorized as “large city,” “southern Sweden,” and “northern Sweden.” Prior to statistical analysis, region of residence was recoded into “large city” and “small city/countryside” from the original categories. Large cities were defined as one of the three largest cities in Sweden (Stockholm, Gothenburg, and Malmö), and “southern Sweden” and “northern Sweden” were recoded into “small city/countryside.”

*Immigrant status* was defined as Yes (born outside Sweden) and No (born in Sweden).

The covariates education and social welfare were selected to capture SES, marital status was a proxy variable for social support and presumed healthcare seeking, region captures proximity to healthcare and immigrant status captures health literacy.

*Chronic obstructive pulmonary disease* (COPD) was used as a proxy variable for smoking and was identified according to ICD-10 diagnosis codes J40-J47.

*Alcohol use disorder* was identified according to ICD-10 F10 and K70 during the study period.

### Statistical analysis

We used Cox regression models to estimate hazard ratios (HRs) with a 95% confidence interval (CI) to test for the association between DUD and incident prostate cancer, fatal prostate cancer, and cancer stage at diagnosis. The men in the cohort were followed up from 1 January 1997 to the first prostate cancer diagnosis, death, migration from Sweden, or 31 December 2016. We used three models: Model 1 was unadjusted; Model 2 was adjusted for age, educational attainment, social welfare, marital status, region of residence, and immigrant status. Model 3 was additionally adjusted for comorbidities (COPD, alcohol use disorder). The proportional hazard assumptions were checked by plotting the incidence rates over time and calculating Schoenfeld (partial) residuals, and no substantial departures from these assumptions were found.

All statistical analyses were performed using SAS version 9.4 (SAS Institute Inc. Cary, NC, USA). All statistical tests were 2-sided and used an *α*-level of 0.05.

### Secondary analyses

A secondary analysis was conducted that repeated the main analyses after restricting the follow-up period to 2003–2016 to enable more complete outpatient ascertainment of DUD prior to prostate cancer diagnosis, since the NPR for outpatient care started in 2001. This study population was 1,493,595.

Another secondary analysis was conducted where we examined the association between DUD and prostate cancer mortality, while restricting to cancer stages I–III and adjusting for cancer stage.

## Results

### Baseline characteristics

The study population included 1,361,532 men aged 50–75 years in 1 January 1997. The average follow-up time was 15.2 ± 5.6 years, with the mean follow-up time for men with and without DUD being 10.1 years and 15.8 years, respectively. The majority (52.2%) had 9 years or less of education, 3.1% had received social welfare, 72.6% were married/cohabiting, 40.4% were living in large cities, and 21.8% were born outside Sweden (Table 1). DUD was identified in 0.7% (*n* = 9,259). Compared with men without DUD, those with DUD were younger, less likely to be married/cohabiting, and more likely to be born in Sweden, live in large cities, receive social welfare, and/or have COPD or alcohol use disorder (Supplementary Table 1).

**Table 1:** Population and number of events of incident and fatal prostate cancer

	Total population		Incident prostate cancer		Fatal prostate cancer	
	No	%	No	%	No	%
Drug use disorders (DUD)						
Non	1,352,273	99.3	129,772	99.3	26,380	99.1
Yes	9259	0.7	891	0.7	236	0.9
Age groups (years)						
50–59	683,095	50.2	58,322	44.6	5174	19.4
60–69	446,555	32.8	49,193	37.6	11,432	43.0
70–75	231,882	17.0	23,148	17.7	10,010	37.6
Educational attainment						
≤ 9 years	710,213	52.2	54,767	41.9	13,557	50.9
10–11 years	243,740	17.9	26,496	20.3	5049	19.0
12 ≤ years	407,579	29.9	49,400	37.8	8010	30.1
Social welfare						
Non	1,319,822	96.9	128,052	98.0	26,138	98.2
Yes	41,710	3.1	2611	2.0	478	1.8
Marital status						
Married/cohabiting	988,709	72.6	96,819	74.1	19,112	71.8
Not married/cohabiting	372,823	27.4	33,844	25.9	7504	28.2
Region of residence						
Large city	549,566	40.4	63,164	48.3	11,610	43.6
Southern Sweden	404,145	29.7	44,715	34.2	9502	35.7
Northern Sweden	407,821	30.0	22,784	17.4	5504	20.7
Immigrant status						
Born in Sweden	1,064,951	78.2	119,511	91.5	24,543	92.2
Born in other countries	296,581	21.8	11,152	8.5	2073	7.8
COPD						
Non	1,261,964	92.7	120,428	92.2	25,015	94.0
Yes	99,568	7.3	10,235	7.8	1601	6.0
Alcohol use disorder						
Non	1,312,518	96.4	127,034	97.2	26,017	97.7
Yes	49,014	3.6	3629	2.8	599	2.3
All	1,361,532		130,663		26,616	

*COPD* chronic obstructive pulmonary disease

### Incident prostate cancer and DUD

In the study population, 9.6% (*n* = 130,663) were diagnosed with prostate cancer. We found no association between DUD and prostate cancer incidence in the univariate analysis (Model 1), nor in the multivariate analysis adjusted for age, educational attainment, social welfare, marital status, region of residence, and immigrant status (Model 2) (Table 2). However, in the multivariate analysis that adjusted for the covariates mentioned above as well as COPD and alcohol use disorder (Model 3), DUD was associated with a slightly increased risk of prostate cancer (HR = 1.07, 95% CI 1.00–1.14, *p* = 0.048). In Model 3, prostate cancer was associated with higher age (HR = 1.41, 95% CI 1.40–1.42), residence in larger cities (HR = 1.27, 95% CI 1.25–1.28), and COPD (HR = 1.04, 95% CI 1.02–1.06). Factors associated with lower risk of prostate cancer included educational attainment < 12 years (HR = 0.82, 95% CI 0.81–0.82), receipt of social welfare (HR = 0.91, 95% CI 0.88–0.95), not being married/cohabiting (HR = 0.94, 95% CI 0.93–0.95), being born outside Sweden (HR = 0.32, 95% CI 0.31–0.32), and alcohol use disorder (HR = 0.86, 95% CI 0.83–0.89).

**Table 2:** Association of drug use disorders (DUD) and incident prostate cancer

Covariates	Model 1				Model 2				Model 3			
	HR	95% CI		*p* value	HR	95% CI		*p* value	HR	95% CI		*p* value
DUD (vs. non)	1.04	0.97	1.11	0.2411	1.03	0.97	1.10	0.3392	1.07	1.00	1.14	0.0476
Age	1.38	1.37	1.39	< 0.0001	1.41	1.40	1.42	< 0.0001	1.41	1.40	1.42	< 0.0001
Educational attainment < 12 years (vs. 12 ≤ years)	0.73	0.72	0.73	< 0.0001	0.82	0.81	0.82	< 0.0001	0.82	0.81	0.82	< 0.0001
Social welfare (vs. no social welfare)	0.69	0.66	0.72	< 0.0001	0.90	0.87	0.94	< 0.0001	0.91	0.88	0.95	< 0.0001
Not married/cohabiting (vs. married/cohabiting)	1.04	1.02	1.05	< 0.0001	0.93	0.92	0.94	< 0.0001	0.94	0.93	0.95	< 0.0001
Region of residence large city (vs. small city/countryside)	1.46	1.45	1.48	< 0.0001	1.27	1.25	1.28	< 0.0001	1.27	1.25	1.28	< 0.0001
Immigrant status (vs. born in Sweden)	0.29	0.29	0.30	< 0.0001	0.32	0.31	0.32	< 0.0001	0.32	0.31	0.32	< 0.0001
COPD (vs. non)	1.19	1.16	1.21	< 0.0001					1.04	1.02	1.06	0.0001
Alcohol use disorder (vs. non)	0.86	0.83	0.89	< 0.0001					0.86	0.83	0.89	< 0.0001

*N* = 1,361,532

*HR* hazard ratio, *CI* confidence interval, *COPD* chronic obstructive pulmonary disease

Model 1: univariate model; Model 2: adjusted for age, educational attainment, social welfare, marital status, region of residence, and immigrant status; Model 3: full adjusted (Model 2 + comorbidities)

### Fatal prostate cancer and DUD

In the study population, 2.0% (*n* = 26,616) died of prostate cancer during the study period. We found an association between DUD and fatal prostate cancer in the univariate analysis (Model 1) (HR = 1.38, 95% CI 1.22–1.57), which persisted and increased slightly after adjustment for age, educational attainment, social welfare, marital status, region of residence, and immigrant status in Model 2 (HR = 1.52, 95% CI 1.34–1.73), and further adjustment for COPD and alcohol use disorder in Model 3 (HR = 1.59, 95% CI 1.40–1.82) (Table 3). In Model 3, fatal prostate cancer was associated with increasing age (HR = 3.12, 95% CI 3.07–3.17), receipt of social welfare (HR = 1.20, 95% CI 1.09–1.31), not being married/cohabiting (HR = 1.17, 95% CI 1.14–1.21), and residence in a large city (HR = 1.07, 95% CI 1.04–1.10). Lower risk of fatal prostate cancer was associated with being born outside Sweden (HR = 0.26, 95% CI 0.25–0.27), COPD (HR = 0.70, 95% CI 0.67–0.74), and alcohol use disorder (HR = 0.90, 95% CI 0.82–0.97). No association was found between educational attainment and fatal prostate cancer.

**Table 3:** Association of drug use disorders (DUD) and fatal prostate cancer

Covariates	Model 1				Model 2				Model 3			
	HR	95% CI		*p* value	HR	95% CI		*p* value	HR	95% CI		*p* value
DUD (vs. non)	1.38	1.22	1.57	< 0.0001	1.52	1.34	1.73	< 0.0001	1.59	1.40	1.82	< 0.0001
Age	3.03	2.99	3.08	< 0.0001	3.11	3.07	3.16	< 0.0001	3.12	3.07	3.17	< 0.0001
Educational attainment < 12 years (vs. 12 ≤ years)	1.05	1.02	1.08	0.0002	1.00	0.98	1.03	0.8965	1.01	0.98	1.04	0.5930
Social welfare (vs. no social welfare)	0.64	0.59	0.70	< 0.0001	1.17	1.06	1.28	0.0010	1.20	1.09	1.31	0.0001
Not married/cohabiting (vs. married/cohabiting)	1.20	1.16	1.23	< 0.0001	1.17	1.13	1.20	< 0.0001	1.17	1.14	1.21	< 0.0001
Region of residence large city (vs. small city/countryside)	1.20	1.17	1.22	< 0.0001	1.06	1.04	1.09	< 0.0001	1.07	1.04	1.10	< 0.0001
Immigrant status (vs. born in Sweden)	0.27	0.26	0.28	< 0.0001	0.27	0.25	0.28	< 0.0001	0.26	0.25	0.27	< 0.0001
COPD (vs. non)	0.92	0.87	0.97	0.0009					0.70	0.67	0.74	< 0.0001
Alcohol use disorder (vs. non)	0.73	0.67	0.79	< 0.0001					0.90	0.82	0.97	0.0083

*N* = 1,361,532

*HR* hazard ratio, *CI* confidence interval, *COPD* chronic obstructive pulmonary disease

Model 1: univariate model; Model 2: adjusted for age, educational attainment, social welfare, marital status, region of residence, and immigrant status; Model 3: full adjusted (Model 2 + comorbidities)

### Prostate cancer stage and DUD

After exclusion of men without data on TNM stage, 1,240,922 individuals remained for the analysis. We found no association between DUD and tumor stage at prostate cancer diagnosis, when adjusting for age, educational attainment, social welfare, marital status, region of residence, immigrant status, COPD, and alcohol use disorder (Table 4).

**Table 4:** Association of drug use disorders (DUD) and prostate cancer stage at time of diagnosis among men with incident prostate cancer

Covariates	Prostate cancer stage I–III (vs. no prostate cancer and prostate cancer stage IV)				Prostate cancer stage IV (vs. no prostate cancer and prostate cancer stage I–III)			
	HR*	95% CI		*p* value	HR*	95% CI		*p* value
DUD (vs. non)	1.00	0.78	1.27	0.9814	0.79	0.11	5.72	0.8119

*N* 1,240,922. Cases of prostate cancer: 10,378

*HR* hazard ratio, *CI* confidence interval, *COPD* chronic obstructive pulmonary disease

*Full adjusted (for age, educational attainment, social welfare, marital status, region of residence, immigrant status, COPD, and alcohol use disorder)

### Secondary analyses

A secondary analysis of incident and fatal prostate cancer with follow-up during 2003–2016 showed that DUD was significantly associated with fatal prostate cancer (HR 2.00; 95% CI 1.71–2.33; *p* < 0.001), when adjusting for age, educational attainment, social welfare, marital status, region of residence, immigrant status, COPD, and alcohol use disorder (Supplementary Table 2).

In a secondary analysis of fatal prostate cancer while restricting to cancer stages I–III and adjusting for cancer stage, DUD was significantly associated with fatal prostate cancer (HR 1.48; 95% CI 1.30–1.69; *p* < 0.001) (Supplementary Table 3).

## Discussion

To our knowledge, this is the first population-based study of prostate cancer incidence, mortality, and stage at diagnosis among men with DUD. We identified DUD as a strong predictor for fatal prostate cancer, which supported our hypothesis regarding mortality, whereas DUD was associated with only a slightly increased risk of incident prostate cancer. Our findings were somewhat similar to the retrospective, longitudinal cohort studies by Chhatre et al. and Jayadevappa et al. that reported that SUD (including alcohol, prescription drugs, or illicit drugs) was associated with higher mortality among men with advanced stage prostate cancer (*n* =  ~ 14,000) [36, 37]. The authors reported that men with ‘drug psychoses and related disorders’ were at particularly high risk of prostate cancer death and all-cause mortality, used more health services (inpatient care, outpatient, and ER visits), and had higher cost of care than men with alcohol dependence or non-dependent use of drugs [36]. In those studies, men with SUD also had higher prostate cancer grade and higher medical comorbidity. While not fully comparable with our study design, these studies showed an increased risk of prostate cancer death among men with DUD that was notable also in our findings.

Our findings persisted after adjustment for previously identified risk factors for prostate cancer incidence and mortality, i.e., age, SES variables [22], and comorbidities related to tobacco smoking and alcohol [30–32]. Even though low SES, tobacco smoking, and excessive alcohol use are all associated with DUD [17, 25–29, 38], these variables could not entirely explain the increased prostate cancer mortality among men with DUD. The increased prostate cancer mortality among men with DUD might, in part, be due to delayed healthcare seeking or poor treatment compliance. This potential explanation is supported by our secondary analysis showing that DUD is associated with fatal prostate cancer, also when excluding men with metastasized cancer (stage IV) and adjusting for stage. Previous research has shown that people with DUD have a high degree of unmet healthcare needs, barriers towards seeking healthcare, and experience of stigma during healthcare encounters [11–13, 39]. This may lead to an overall poorer health, which may increase mortality due to specific causes, such as prostate cancer. People with DUD also under-utilize cancer screening [18–21]. DUD is further often associated with psychiatric comorbidity [15–17]. Tuesley et al. reported low frequency of prostate cancer screening among men with severe mental illness, and suggested that screening non-participation “may explain some of the mismatch between cancer incidence and mortality in people with severe mental illness” [40]. Fried et al. found that serious mental illness in men with non-metastatic prostate cancer was associated with receipt of less aggressive treatment and worse cancer-specific survival [41]. In Sweden, PSA testing is performed on the individual’s own initiative and may be under-utilized by men with DUD. Self-rated genital and lower urinary tract symptoms were prevalent in 47% of a sample of Swedish people in opioid substitution treatment (OST), but a minority (20%) reported having sought healthcare for these problems [11].

In addition to our main results, we found that educational attainment < 12 years, having received social welfare, and not being married/cohabiting were inversely associated with incident prostate cancer, but positively (social welfare, marital status) or non-significantly (educational attainment) associated with fatal prostate cancer. These findings are in line with the previous research identifying an association between high SES and higher incidence of low- to moderate-risk prostate cancer, possibly related to increased screening [42, 43]; whereas low SES, including income, marital status, and educational attainment, has been associated with poorer prostate cancer outcomes [44–47]. Being a resident in a large city was associated with both incident and fatal prostate cancer, while being born outside Sweden was inversely associated with both incident and fatal prostate cancer. The latter finding was consistent with the results from several studies showing lower prostate cancer incidence and mortality among immigrants in, e.g., Australia, Germany, and Canada [48–50]. While incident prostate cancer was associated with COPD but inversely associated with alcohol use disorder, both COPD and alcohol use disorder were inversely associated with fatal prostate cancer, which might be explained by high fatality rates related to COPD and excessive alcohol use, such as increased mortality in liver diseases and other alcohol-related diseases. It is also notable that COPD is not equivalent to tobacco smoking, and some people diagnosed with COPD might be more likely to quit smoking than people without COPD. This could potentially affect the risk of prostate cancer.

Our finding that men with DUD were at high risk for fatal prostate cancer has important clinical implications. Improved access to and retention in OST has led to an aging population of patients with treated opioid dependence [51], and thus reaching ages when prostate cancer is more common. Our findings suggest that healthcare professionals and policy-makers need to be aware of the increased health risks in men with DUD in order to improve prostate cancer screening, treatment, and survival in this population. Future research regarding healthcare seeking barriers and prostate cancer treatment compliance among men with DUD is needed in order to decrease these health inequities.

### Strengths and limitations

The nationwide data used in this study were obtained from several high-quality national registers. The Swedish Cancer Register has almost 100% validity and coverage [52–54], and 85–95% of the diagnoses in the NPR are valid [55]. The Swedish Total Population Register is nearly 100% complete [56, 57].

The broad definition of DUD applied in this study, ranging from a diagnosis in the NPR to a registration of drug possession in the Suspicion Register, has been used in several previous studies [7, 58]. Although this definition may include some men with sporadic drug use rather than DUD, our intention was to include men with DUD that had not been noted by the healthcare system and registered in the NPR. According to a survey made in 2017 by the Swedish Council for Information on Alcohol and Other Drugs, an estimated 1.8% of the Swedish adult population reported drug use [59]. The 0.7% prevalence of DUD from our register data is lower than the survey data, which likely is due to the high age (50–75 years) at inclusion in our study.

The association between DUD and fatal prostate cancer had a higher HR in the secondary analysis beginning in 2003 than in the main analysis (HR 2.00 vs. HR 1.59). This might be explained by some men with DUD being misclassified as not having DUD in the main analysis (before outpatient data were available), thus resulting in underestimation of the HR for fatal prostate cancer associated with DUD.

Data on types of substances used, quantification of DUD, or addiction treatment, would have allowed for more refined analyses. Severe DUD (compared to occasional, recreational drug use) might be associated with higher cancer mortality due to lower healthcare seeking and less adequate preventive healthcare, but could also be associated with lower cancer mortality due to premature, drug-related death [3, 4]. Given the limitations of the registers used, we did not have access to such data on a total-population level. Main drug and DUD severity in relation to prostate cancer incidence and mortality would be useful to examine in future research. Such research could also assess potential biological mechanisms behind use of specific drugs and prostate cancer incidence and mortality. Information on receipt of PSA tests, PSA level at diagnosis, prostate cancer grade, and time between diagnosis and treatment was also unavailable for our analyses. Although we found no association between DUD and prostate cancer stage, it is still possible that DUD is associated with more advanced disease at diagnosis, which is important to assess further in future studies. Our findings on prostate cancer stage at diagnosis should be interpreted with caution since data on cancer stage wer available only in 7.9% of the sample with incident prostate cancer.

## Conclusion

In this large national cohort of men, we found that DUD was associated with slightly higher prostate cancer incidence and substantially higher prostate cancer mortality. Our hypothesis that DUD would be associated with an increased prostate cancer mortality was supported by the analyses, while the hypothesis that DUD would be associated with higher prostate cancer stage at the time of diagnosis was not. Our findings should raise attention among clinicians and health policy-makers towards a disadvantaged group of men in need of easily accessible prostate cancer evaluation and treatment.

## Supplementary Information

Below is the link to the electronic supplementary material.

Supplementary file1 (DOC 202 KB)

## Data Availability

The data used to support the findings of this study are restricted by the Swedish authorities, in order to protect people’s privacy. Please refer to Kristina Sundquist for questions on data availability.
