# Supplementary material for: Drug use disorder and risk of incident and fatal prostate cancer among Swedish men: a nationwide epidemiological study
Source: Cancer Causes Control. 2021 Nov 7;33(2):213–22. doi: 10.1007/s10552-021-01513-2 (PMC8776671; doi:10.1007/s10552-021-01513-2)
Supplement: Supplementary file 1 — Supplementary file1 (DOC 202 KB) [file 10552_2021_1513_MOESM1_ESM.doc]

**Supplementary material**

**Supplementary Table 1. Population and number of events of drug use disorders (DUD). N=1,361,532.**

|  | **No DUD** | |  | **DUD** | |
| --- | --- | --- | --- | --- | --- |
|  | **No.** | **%** |  | **No.** | **%** |
| Total population | 1352273 |  |  | 9259 |  |
| Age groups (years) |  |  |  |  |  |
| 50-59 | 677017 | 50.1 |  | 6078 | 65.6 |
| 60-69 | 444287 | 32.9 |  | 2268 | 24.5 |
| 70-75 | 230969 | 17.1 |  | 913 | 9.9 |
| Educational attainment |  |  |  |  |  |
| ≤ 9 years | 706149 | 52.2 |  | 4064 | 43.9 |
| 10-11 years | 241341 | 17.8 |  | 2399 | 25.9 |
| 12≤ years | 404783 | 29.9 |  | 2796 | 30.2 |
| Social welfare |  |  |  |  |  |
| Non | 1312031 | 97.0 |  | 7791 | 84.1 |
| Yes | 40242 | 3.0 |  | 1468 | 15.9 |
| Marital status |  |  |  |  |  |
| Married/cohabiting | 984400 | 72.8 |  | 4309 | 46.5 |
| Not married/cohabiting | 367873 | 27.2 |  | 4950 | 53.5 |
| Region of residence |  |  |  |  |  |
| Large city | 544385 | 40.3 |  | 5181 | 56.0 |
| Southern Sweden | 401397 | 29.7 |  | 2748 | 29.7 |
| Northern Sweden | 406491 | 30.1 |  | 1330 | 14.4 |
| Immigrant status |  |  |  |  |  |
| Born in Sweden | 1057033 | 78.2 |  | 7918 | 85.5 |
| Born in other countries | 295240 | 21.8 |  | 1341 | 14.5 |
| COPD |  |  |  |  |  |
| Non | 1254394 | 92.8 |  | 7570 | 81.8 |
| Yes | 97879 | 7.2 |  | 1689 | 18.2 |
| Alcohol use disorder |  |  |  |  |  |
| Non | 1306474 | 96.6 |  | 6044 | 65.3 |
| Yes | 45799 | 3.4 |  | 3215 | 34.7 |
| Incident prostate cancer |  |  |  |  |  |
| Non | 1222501 | 90.4 |  | 8368 | 90.4 |
| Yes | 129772 | 9.6 |  | 891 | 9.6 |
| Fatal prostate cancer |  |  |  |  |  |
| Non | 1325893 | 98.0 |  | 9023 | 97.5 |
| Yes | 26380 | 2.0 |  | 236 | 2.5 |
| Prostate cancer stage |  |  |  |  |  |
| Stage I | 609 | <0.1 |  | 3 | <0.1 |
| Stage II | 7449 | 0.6 |  | 48 | 0.5 |
| Stage III | 2066 | 0.2 |  | 15 | 0.2 |
| Stage IV | 187 | <0.1 |  | 1 | <0.1 |
| Cancer stage unknown | 119461 | 8.8 |  | 824 | 8.9 |
|  |  |  |  |  |  |
|  |  |  |  |  |  |
|  |  |  |  |  |  |
| COPD=Chronic obstructive pulmonary disease. | |  |  |  |  |

**Supplementary Table 2. Association of drug use disorders (DUD) and incident and fatal prostate cancer, follow-up 2003–2016. N=1,493,595.**

|  | **Incident prostate cancer** | | | |  | **Fatal prostate cancer** | | | |
| --- | --- | --- | --- | --- | --- | --- | --- | --- | --- |
| **Covariates** | **HR*** | **95% CI** | | **P-value** |  | **HR*** | **95% CI** | | **P-value** |
| DUD (vs. non) | 0.94 | 0.87 | 1.01 | 0.0739 |  | 2.00 | 1.71 | 2.33 | <.0001 |
| Age | 1.49 | 1.48 | 1.50 | <.0001 |  | 3.44 | 3.36 | 3.52 | <.0001 |
| Educational attainment <12 years (vs. 12≤ years) | 0.79 | 0.78 | 0.80 | <.0001 |  | 1.06 | 1.02 | 1.10 | 0.0014 |
| Social welfare (vs. no social welfare) | 0.85 | 0.81 | 0.90 | <.0001 |  | 1.31 | 1.14 | 1.50 | <.0001 |
| Not married/cohabiting (vs. married/cohabiting) | 0.93 | 0.92 | 0.94 | <.0001 |  | 1.19 | 1.15 | 1.24 | <.0001 |
| Region of residence large city (vs. small city/countryside) | 1.27 | 1.26 | 1.29 | <.0001 |  | 1.04 | 1.01 | 1.08 | 0.0125 |
| Immigrant status (vs. born in Sweden) | 0.33 | 0.32 | 0.34 | <.0001 |  | 0.31 | 0.29 | 0.33 | <.0001 |
| COPD (vs. non) | 1.12 | 1.10 | 1.15 | <.0001 |  | 0.86 | 0.80 | 0.92 | <.0001 |
| Alcohol use disorder (vs. non) | 0.91 | 0.88 | 0.94 | <.0001 |  | 0.86 | 0.77 | 0.96 | 0.0080 |

HR=Hazard ratio. CI=Confidence interval. COPD=Chronic obstructive pulmonary disease.

*: Full adjusted (for age, educational attainment, social welfare, marital status, region of residence, immigrant status, and comorbidities)

**Supplementary Table 3. Association of drug use disorders (DUD) and fatal prostate cancer after adjustment for cancer stage and exclusion of individuals with prostate cancer stage IV. N=1,361,344.**

|  | **Fatal prostate cancer** | | | |
| --- | --- | --- | --- | --- |
| **Covariates** | **HR*** | **95% CI** | | **P-value** |
| DUD (vs. non) | 1.48 | 1.30 | 1.69 | <0.0001 |
|  |  |  |  |  |

HR=Hazard ratio. CI=Confidence interval. COPD=Chronic obstructive pulmonary disease.

*: Full adjusted (for tumor stage, age, educational attainment, social welfare, marital status, region of residence, immigrant status, and comorbidities)
